# Visual communication via the design of food and beverage packaging

**DOI:** 10.1186/s41235-022-00391-9

**Published:** 2022-05-12

**Authors:** Charles Spence, George Van Doorn

**Affiliations:** 1 Crossmodal Research Laboratory, Oxford University, Oxford, OX2 6GG UK; 2 School of Science, Psychology and Sport, Churchill Campus, Federation University Australia, Churchill, VIC 3842 Australia; 3 Health Innovation and Transformation Centre, Mt Helen Campus, Federation University Australia, Ballarat, VIC 3350 Australia; 4 Successful Health for At-Risk Populations (SHARP) Research Group, Mt Helen Campus, Federation University Australia, Ballarat, VIC 3350 Australia

**Keywords:** Visual packaging design, Food and beverage, Crossmodal correspondences

## Abstract

A rapidly growing body of empirical research has recently started to emerge highlighting the connotative and/or semiotic meanings that consumers typically associate with specific abstract visual design features, such as colours (either when presented individually or in combination), simple shapes/curvilinearity, and the orientation and relative position of those design elements on product packaging. While certain of our affective responses to such basic visual design features appear almost innate, the majority are likely established via the internalization of the statistical regularities of the food and beverage marketplace (i.e. as a result of associative learning), as in the case of round typeface and sweet-tasting products. Researchers continue to document the wide range of crossmodal correspondences that underpin the links between individual visual packaging design features and specific properties of food and drink products (such as their taste, flavour, or healthfulness), and the ways in which marketers are now capitalizing on such understanding to increase sales. This narrative review highlights the further research that is still needed to establish the connotative or symbolic/semiotic meaning(s) of particular combinations of design features (such as coloured stripes in a specific orientation), as opposed to individual cues in national food markets and also, increasingly, cross-culturally in the case of international brands.

## Introduction

The visual design of food and beverage product packaging is at something of a crossroads. The field currently lies between the traditional art and design approach—often based on the intuitions of creative designers/marketers (and/or the results of focus groups or in-depth interviews; Cheskin, 1957, 1967, 1972; Lunt, 1981; Rapaille, 2007; Stern, 1981)—and the more scientific approach to visual communication (i.e. presenting information graphically, such that it creates meaning concerning the product and its attributes/brand associations; Underwood, 1993, 1999; Underwood & Klein, 2002; Underwood & Ozanne, 1998; Underwood et al., 2001). The latter approach is increasingly coming to be based on our growing understanding of, for example, the crossmodal correspondences (Spence, 2011, 2012; Velasco & Spence, 2019a; Velasco et al., 2016b; cf. Batra et al., 2016; Schifferstein et al., 2013; Skaczkowski et al., 2016; Thomson, 2016).

Crossmodal correspondences refer to the tendency for a feature or attribute in one sensory modality (e.g. the colours pink and red) to be associated with a sensory feature in another sensory modality (e.g. a sweet taste; Ngo et al., 2013; Spence & Parise, 2012; Woods et al., 2013). Often, these connections between the senses are surprising, much like synaesthesia.^1^ Indeed, some researchers have even suggested that synaesthetic inducer-concurrent relations could be used productively in the field of product design (Haverkamp, 2014) and/or product packaging/marketing (cf. Crisinel & Spence, 2012). That said, it is important to stress that the approach outlined here, based on crossmodal correspondences, differs from the phenomenon of synaesthesia in that the cross-sensory connections expressed in the former case tend to be shared between people, whereas synaesthesia is defined by the idiosyncratic nature of the inducer-concurrent mapping (see Deroy & Spence, 2013; Spence, 2019).

Visual design features are not only associated with taste/flavour attributes,^2^ but with a range of connotative and semantic meanings (e.g. green = healthy) as typically assessed by research using the semantic differential technique (e.g. Morich, 1981; Snider & Osgood, 1969; see also Kunz et al., 2020). However, design cues (such as colour) are also used to set consumer expectations around product variant, brand, quality, and price (with black packaging often linked with luxury and premiumness, whereas orange is typically associated with cheapness; see Velasco & Spence, 2019c; Wheatley, 1973). Given that we typically see colour in context (Elliot & Maier, 2012), and that context is (at times) influenced by culture, it might be thought that it would be unlikely for there to be many universal meanings associated with specific visual design features, such as a particular hue. That said, Tham et al. (2020) recently tested English monolinguals, Chinese bilinguals, and Chinese monolinguals in order to establish the conceptual associations that the different groups had with colour words and colour patches. According to their results, white was associated with purity, blue was related to water/sky themes, green was linked to healthy, purple was regal, and pink was linked to female for all three groups. At the same time, however, red and orange were associated with enthusiastic in Chinese, whereas red was associated with attraction in English. In other words, Tham et al.’s results highlight the existence of both a number of cross-cultural similarities and differences in the conceptual associations that different groups of people appear to hold with colours and colour words.

In this narrative review, and in relation to visual design, we are particularly interested in the crossmodal correspondences that may exist between various ‘abstract’ visual features^3^—colours (either when presented individually or in combination), simple shapes/curvilinearity, and the orientation and relative position of those design elements on the packaging—and the chemical senses (specifically taste/flavour). That said, several other connotative/symbolic/semantics associations of visual features/attributes (e.g. with healthy/natural, price, premiumness, etc.) will also be discussed (see Marques da Rosa et al., 2019). It is important to stress here that the term ‘abstract’ here refers to those features that are not associated with a specific object—while many abstract visual design features can be classed as simple stimuli, some patterns and face-like arrangements of lines might be considered complex. Hence, a patch of blue or a specific simple shape like a circle can be considered abstract design features, whereas the picture or outline of a hamburger, say, or the image of some fruit (see Piqueras-Fiszman et al., 2013), would not.

### Visual design of product packaging based on crossmodal correspondences

While a handful of famous designers and marketers have long been lauded for their design choices that helped boost long-term brand/product success (see Cheskin, 1957, 1967, 1972; Dichter, 1975; Favre & November, 1979; Graham, 2016), it often appeared as though their decisions were based on intuition, sometimes backed-up by the results of consumer/focus-group research and in-depth interviews (Catterall & Maclaran, 2006; Lunt, 1981; Samuel, 2010). By contrast, an emerging body of empirical research on the crossmodal correspondences is now starting to help establish the connotative meaning of a variety of different abstract visual design features. In particular, a broad array of findings from experimental psychology have helped to establish the meanings (connotative and otherwise) that are associated with (or primed by) everything from colours (Déribéré, 1978; Ho et al., 2014; Spence, 2020a; Van Doorn et al., 2014) to shapes (Dichter, 1971; Mirabito et al., 2017; Motoki & Velasco, 2021; Spence, 2012, 2020b; Spence & Van Doorn, 2017; Van Doorn et al., 2017; Velasco & Spence, 2019a; see also Yarar et al., 2019), and from curvilinearity to the relative position of various design elements on product packaging (Romero & Biswas, 2016; Simmonds et al., 2018b; Sundar & Noseworthy, 2014; Velasco et al., 2015c).

Several of these visual cues, such as a curved line that, at least when presented horizontally, can be interpreted as a smile (Karim et al., 2017; Salgado-Montejo et al., 2015b; cf. Kühn et al., 2014; Windhager et al., 2008) and patterns that may be interpreted as looking like a snake, spider, or scorpion (Hoehl et al., 2017; Isbell, 2006; Van Lee et al., 2013; LoBue, 2014; Spence, 2021a) have been associated with possibly innate responses that are (often) attention-capturing, albeit typically negatively valenced in the latter cases. However, the meaning of many other visual design cues is much more likely to be established on the basis of associative learning.^4^ Here it is also important to consider the commonly accepted symbolic and semiotic meaning of packaging design features in the food and beverage marketplace (e.g. cartoon portrait logos and their association, in Western cultures, with their often humorous messages; Barthel, 1989; Danesi, 2013; Garber et al., 2008; Gardner & Levy, 1955; Levy, 1959; Mick, 1986; Plasschaert, 1995).

Researchers have, for example, highlighted how the packaging of flavours/varieties of crisps/potato chips tend to be coloured in a specific (albeit somewhat arbitrary) manner (see Piqueras-Fiszman et al., 2012). Green packaging, for example, is often (though not always) associated with cheese and onion flavour in the UK, whereas blue packaging typically signals salt and vinegar instead (though see Visser, 2009, p. 109, on the suggestion that salt and vinegar type crisps are colour-coded purple-pink). As another example, one might take the different colour associations with full-, half-, and low-fat milk that exist in different parts of the world (Simmonds & Spence, 2019). In Australia, for example, light milk (i.e. 2% milk) is often tagged with colour-coded caps that are light blue, while full-cream milk often has dark blue caps and labels (Cutolo, 2021). By contrast, in the UK, skimmed milk (containing less than 0.3% fat) is typically tagged with red caps, semi-skimmed milk (i.e. less than 2% fat) is tagged with green, and full-fat milk is tagged with the colour blue.

Different packaging label colours are sometimes also associated with different forms of animal protein (e.g. consider the different colour codes that are often used to help distinguish lamb, beef, poultry, and pork) in the fresh meat category (see Simmonds & Spence, 2019, for a review). As such, a particular hue may be associated with a range of different attributes/qualities, and the extent to which different associations are primed may well ultimately depend on the context in which that colour happens to be presented (see Elliot & Maier, 2012; Motoki & Velasco, 2021), and the familiarity of the consumer with the conventions of the marketplace in which they happen to find themselves.

**Fig. 1:**
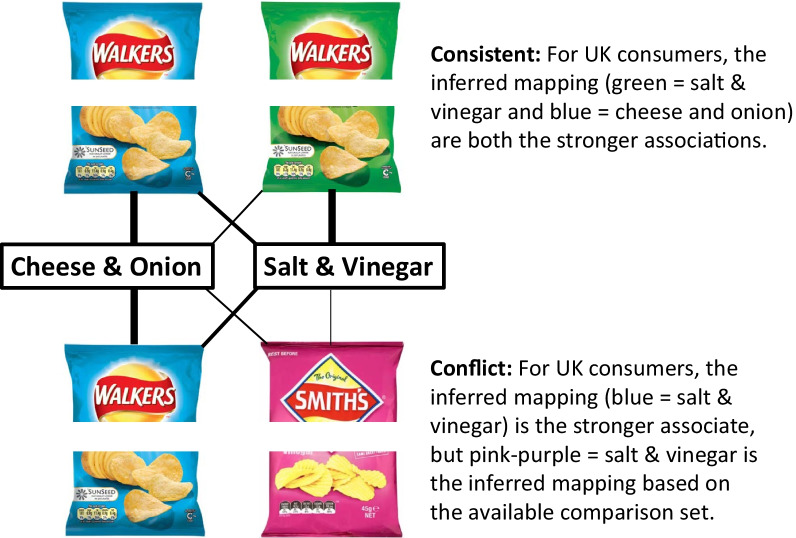
Distinction between colour–flavour associations and inferred mappings, showing colour–flavour association strengths for flavours ‘Cheese and onion’ and ‘Salt and vinegar’ with crisp packaging colours blue, green, and pinkish-purple (thicker lines connecting flavours with colours indicate stronger associations). What such a hypothetical situation highlights is how the colour–flavour mapping may result from inference rather than direct association. Figure adapted from Schloss et al. (2018)

Such market-/country-specific differences also speak to the role of culture (built on habit and prior experience/exposure) in determining the meaning of colour in a given context (see also Jonauskaite et al., 2019a). It is important to stress, though, that in contrast to the often-published observations of those in marketing who, over the years, have attempted to map out the abstract meaning of colours (e.g. Aslam, 2006; Jacobs et al., 1991; Wheatley, 1973), or emotional associations with colours (e.g. Adams & Osgood, 1973), colour is nearly always seen in context (Elliot & Maier, 2012; though see also Amsteus et al., 2015). Furthermore, researchers have recently argued for the importance of context in terms of a theory of semantic discriminability (Mukherjee et al., 2022; Schloss et al., 2021). According to the latter researchers, the mapping of a colour to a particular concept is often inferred on the basis of other stimuli in the comparison group, rather than being based directly on the strength of the underlying association.^5^ Figure 1 frames Mukherjee and colleagues’ distinction in the context of the colour of potato chip packaging. What their theory means, in practice, is that sometimes the inferred colour–flavour mapping need not necessarily reflect the strongest colour–flavour association.

Various abstract visual design features normally exist on product packaging alongside semantic information concerning brand name, product description, (any) product imagery, and/or possibly also serving suggestions (Rebollar et al., 2012; Simmonds & Spence, 2019; Thomson, 2016; Visser, 2009). Although product, and other kinds of, visual imagery seen on packaging (or seen through transparent windows in the packaging) undoubtedly play an important role in determining the consumer’s impressions of a variety of food and beverage products (Simmonds & Spence, 2017, 2019; Simmonds et al., 2018a), reviewing the literature documenting the role played by such concrete (typically semantically meaningful) visual cues falls beyond the scope of this targeted narrative review. This review will instead focus specifically on abstract visual design features and their relation to product taste/flavour, healthiness, price, etc. Those readers interested in the influence of product imagery are directed to Simmonds and Spence’s (2019) review.

### Review outline

In ‘On the meaning associated with individual abstract visual packaging cues’ section, we review what is currently known about the connotative meanings (including crossmodal correspondences) associated with specific design features such as colour, shape, orientation/position, and the use of convention-defying visual designs. In ‘Combining abstract visual design features’ section, the discussion is extended to the meaning of combinations of abstract visual cues using, as a recent commercial example, the under-researched combination of colour and stripes (i.e. a band of colour that differs from the colour on either side of it). Applied researchers have now deconstructed a number of elements of food and beverage packaging design in order to try and discern how to optimize everything from the connotation of ‘healthiness’ (Cavallo & Piqueras-Fiszman, 2017; Huang & Lu, 2013, 2015; Marques da Rosa et al., 2019; Reinoso-Carvalho et al., 2021), spiciness (Gil-Pérez et al., 2019), and quality (Pombo & Velasco, 2021; Wang, 2013). To date, however, only limited research has investigated the influence of vertical or horizontal orientation on consumer perception and product sales. This is demonstrated by the fact that in recent books on packaging (e.g. Velasco & Spence, 2019a, b), there is virtually no mention of the topic. As such, there is a need to review the existing literature and make recommendations for future research. The ‘Conclusions and future directions’ section offers some directions for future research. Areas that are not covered by this review include the consumers’ response to innovations in specific packaging design/technology, nor will issues related to the sustainability of product packaging be discussed (e.g. Associated Press, 2013; Azzi et al., 2012; Rundh, 2005; Silayoi & Speece, 2007).

Note that in addition to providing an up-to-date review of the literature on visual aspects of packaging design, we also highlight several further concrete areas for future packaging research. These include determining which of the many meanings associated with specific abstract design features such as colours or packaging shapes are primed in the mind of the consumer under everyday conditions (i.e. away from the specific task constraints typically imposed by the experimenter in most laboratory research). Having determined several different meanings that are associated, individually, with specific visual design features, further research is clearly also needed to help determine which cues dominate and/or how different abstract design features combine to convey specific meanings to consumers in different markets/contexts (Visser, 2009). A priori, one might consider whether sub-/super-/additive interactions will be observed when various visual design cues (e.g. colour and shape) are combined. Alternatively, however, it would also seem possible that one cue, such as colour might tend to dominate over other cues (such as, for example, colour dominating over shape, typeface, or texture). At the same time, however, it is also important to stress the fact that the intramodal perceptual grouping (Wagemans, 2015) of visual cues may give rise to a different meaning/association entirely than that associated with, or primed by, the individual sensory cues (see Dreksler & Spence, 2019).

Over the years, a number of different theoretical accounts have been put forward in order to try and explain the meanings/associations that may be primed by different visual design features (see Table 1 for a summary of the various accounts that have been used to help explain the meaning of abstract visual design cues). The accounts include (a) grounded cognition theory where, for example, a ‘strong is heavy’ metaphor is activated, and thus congruency dictates that heavy objects should appear at the bottom of packaging (Fenko et al., 2018), (b) conceptual metaphor theory where healthy foods are associated with high verticality, and thus should be situated at the top of product packaging (Wang & Basso, 2021), (c) the connotative meaning account based on the semantic differential technique (see Table 2 for a summary of the various different methods used by researchers in this area), and crossmodal correspondences (e.g. green = healthy; Morich, 1981), (d) the theory of semiotics where signs convey meaning (e.g. cartoon portrait logos mentioned above; Barthes, 1977; Chandler, 2017), and (e) various evolutionary explanations where stripes may have evolved to attract attention. Ultimately, in terms of parsimony, it would obviously be desirable to consider whether any unifying explanatory account might be invoked/developed to help provide an overarching explanation for the meaning of visual design. However, when we take a careful look at each visual design cue in turn (see below), there is as yet little progress in developing such a commonly agreed account of visual design.

**Table 1:** Summary of various meanings of abstract visual design cues in product packaging

	Abstract visual design cue in product packaging			
	Colour	Shape (orientation)	Texture (and material properties)	Stripes (and position/elevation)
Crossmodal correspondences	Colour-taste mappings (e.g. pinkish-red = sweet; blue and white = salt; Spence et al., 2015b); Colour-pairs = taste (Woods et al., 2016)	Shape-taste mappings (e.g. round = sweet; sour = angular; Spence & Deroy, 2013; Turoman et al., 2018; Velasco et al., 2016a, b)	Textures incorporating rounded elements = sweet (Barbosa Escobar et al., 2020) but more research needed	Elevation-taste mappings (e.g. sweet higher than bitter: Velasco et al., 2019a, b; cf. Sunaga et al., 2016)
Connotative meaning	Hue-connotative meaning (e.g. black = passive, bad, and strong; grey = passive, bad, and weak; white = good and weak; red = strong; yellow = weak; green and blue = good; Adams & Osgood, 1973)	Shape-connotative meaning (e.g. round = pleasant; triangle = strong; Cheskin, 1981). Linear element ascending to right = success (see Spence et al., 2019, for a review)	Data not available though presumably shiny/ metallic associated with premium (see Spence, 2021b, for a review)	Vertical stripes = luxury (Van Rompay et al., 2012, 2019; Wang & Basso, 2021)
Symbolic meaning	Purple = funereal in Japan; Orange = cheap; Black = luxury/premium (Jacobs et al., 1991; Spence & Velasco, 2019; Tham et al., 2020; Wheatley, 1973)	Tall and thin packaging = diet product (Raghubir & Greenleaf, 2006) cf. Cheskin, 1951, p. 110–111); Line ascending to right = success (cf. Spence et al., 2019)	Data not available; though presumably shiny/metallic associated with premium (Spence, 2021b)	Elevation and power (Sundar & Noseworthy, 2014); Laterality and healthiness (Romero & Biswas, 2016)
Semantic meaning	Signature brand colours (e.g. Dairy Milk purple; Baxter et al., 2018; Bowcott, 2013); Crisp packet colours signifying flavour/brand (Piqueras-Fiszman & Spence, 2012)	Image mould (e.g. Coke contour bottle (Anon., 1994); Wishbone salad dressing (Meyers, 1981); Listerine (Parise & Spence, 2012); or bamboo bottle (Visser, 2009)	Product texture (e.g. packaging with fruit-like texture; or Velvety toilet paper prime associated semantic meaning; Spence, 2019b)	When combined with colour, stripes take on semantic meaning (e.g. Cornishware; LGBQ Rainbow stripes; Yates, 2021)
Evolutionary account	Red = sexually receptive and arousing (e.g. Changizi et al., 2006; Humphrey, 1976; Pazda et al., 2011), but colour cues also key in foraging (Foroni et al., 2016; Sumner & Mollon, 2003)	Shape of danger (e.g. snake/spider-like; LoBue, 2014; Spence, 2021a); Seemingly innate affective response to orientation (see Karim et al., 2016)	Certain (slimy) textures associated with off-food therefore avoided; shiny textures look like water and so liked (Spence, 2021b)	When combined with colour may indicate natural danger (e.g. bees, snakes, etc.; (Coborn, 1991; Lieske & Myers, 1994), or camouflage

**Table 2:** Methods used to assess the nature and/or strength of the expectations and associations that people/consumers hold with specific visual features in product packaging

Technique	Description	Representative study
Focus group/in-depth interviewing	Traditional approach to eliciting insights concerning the consumer's associations. Little standardization in terms of approach	Questionable scientific validity of such traditional approaches popularized by famous marketers (e.g. see Cheskin, 1957, 1967, 1972; Lunt, 1981; Rapaille, 2007; Stern, 1981)
Word analysis (WA)	Excellent technique to reveal spontaneous top of mind associated with packaging	Piqueras-Fiszman et al. (2013) used WA together with eye-tracking to assess the associations with variety of visual design choices for packaging for jam/marmalade bottle
Implicit association task (IAT)	Robust experimental technique capable of assessing the strength of people's associations with specific packaging attributes	Parise and Spence (2012) used IAT to assess strength of association between angularity of packaging silhouette (i.e. image mould) and expected strength/efficacy of contents
Semantic differential technique (SDT)	Longstanding approach that helps researchers to determine the connotative associations with packaging exemplars	Henson et al. (2006) used SDT to assess connotative associations with a variety of visual shampoo bottle designs (cf. Kunz et al., 2020; Morich, 1981; Schaefer & Rotte, 2010)
Conjoint analysis (CA)	Excellent technique to assess the relative strength of different associations with a variety of packaging design solutions	Baptista et al. (submitted) used CA to assess the relative importance of colour vs. texture to chocolate packaging (cf. Ares & Deliza, 2010; Gislason et al., 2020)
Temporal dominance of sensations (TDS)	Contemporary analysis technique that enables researchers to track the changing associations with packaging over time (typically 10 s of secs)	Merlo et al. (2018) used TDS to track impact of hamburger packaging colour on consumers' emotions. See also Schifferstein et al. (2013), for temporal assessment over lifetime of consumer's interaction with product
Neuroimaging (e.g. ERP, fMRI)	Little used to date, but various neuroimaging techniques help researchers to understand neural mechanisms underpinning behaviour	Huang et al. (2021) used fMRI to assess neural consequences of colour-taste incongruency in packaging design (cf. Moya et al., 2020). Weinstein (1981) for an early attempt to use ERP

**Fig. 2:**
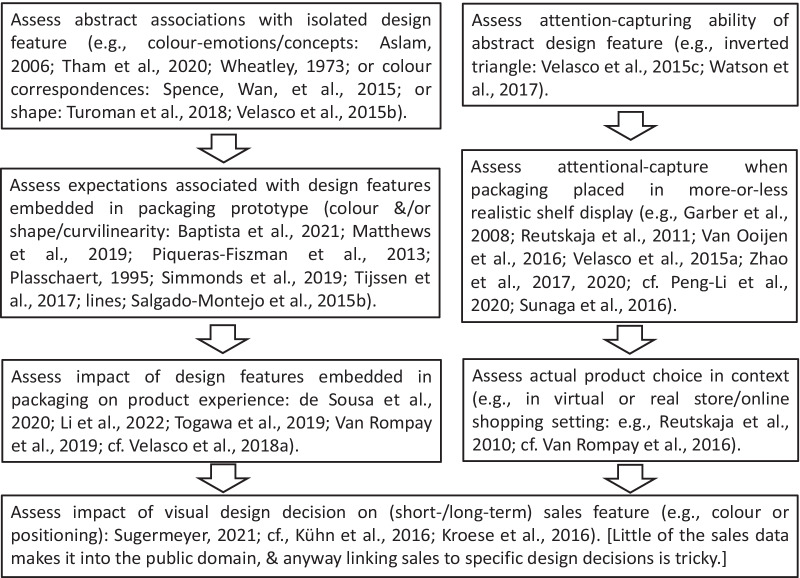
Assessment of visual design choices regarding specific individual design features (e.g. use of a particular colour or shape) at various stages of the packaging (design) journey

As highlighted in Fig. 2, it is clear that there are multiple roles for visual design cues, both related to communicating meaning, or setting expectations, as well as in terms of attentional capture in a realistic visual (multisensory) environment (e.g. Peng-Li et al., 2020). Certainly, there is interest in those factors that facilitate attentional selection (Reutskaja et al., 2011). Here, it is worth stressing that visual design of product packaging has not only been shown to set specific expectations but can also modify people’s product experience. Very often, the approach used by researchers in this area is first to establish the expectations that are primed in the mind of the consumer on being presented with specific packaging designs. Thereafter, on occasion, researchers will then assess whether the differing expectations set by different packaging designs carry through to influence the consumer’s experience of the product itself (de Sousa et al., 2020; Togawa et al., 2019; Van Rompay et al., 2019; cf. Carvalho & Spence, 2019).

Ultimately, of course, the role of effective product packaging is not solely to communicate with the consumer and, on occasion, to enhance product experience, packaging also plays an important role in capturing the consumer’s attention on the shelf or online product display (see Fig. 2). It is intriguing to note here how a distinct body of research has attempted to assess the effectiveness of attentional capture, and the ease of finding a given target product on a more or less realistic shelf/online display (Reutskaja et al., 2011; Zhao et al., 2017). Ultimately, of course, the success of packaging designs is reflected in long-term sales, though here there simply tends to be less publically available research (Sugermeyer, 2021; cf. Kroese et al., 2016; Kühn et al., 2016).

## On the meaning associated with individual abstract visual packaging cues

In this section, we review the evidence concerning the various meanings that may be associated with specific abstract visual cues in the context of product packaging (focusing primarily on the case of food and beverage packaging). Here, the focus will be on the meanings that consumers associate with colours, basic shapes, visual textures (Barbosa Escobar et al., 2020; see also Matthews et al., 2019), as well as the orientation and relative position of specific design elements. At the outset, it is worth highlighting the fact that there are different denotative, connotative, semiotic, and semantic meanings potentially associated with specific abstract visual design features, either when presented individually, or more commonly, when presented in combination (see Visser, 2009). That is, abstract visual design features may be associated with a specific product, brand, or category of product (see Baxter et al., 2018). Abstract visual design features such as colour or shape may also come to be associated with other product attributes such as healthiness, naturalness, indulgence, luxury, or cheapness (see Cavallo & Piqueras-Fiszman, 2017; Mai et al., 2016; Piqueras-Fiszman et al., 2012; Schuldt, 2013; Tijssen et al., 2017; Velasco & Spence, 2019c, for examples). There is also a ‘green/environmental concern’ association with unsurprisingly, the colour green (see Schloss et al., 2018, in the context of recycling).

The focus in this review will primarily be on trying to understand the ‘meaning’ of various different abstract visual design features in terms of the crossmodal correspondences that have been established with sensory properties of the food and beverage products themselves, such as sweetness. At the same time, however, we will also summarize the relevant literature on the connotative meanings of abstract visual design features, such as active–passive, good-bad, dominant-submissive, that have been established by research using the semantic differential technique (Adams & Osgood, 1973; Henson et al., 2006; Osgood et al., 1957). Over the years, Word Association (Piqueras-Fiszman et al., 2013), Implicit Association Tests (Parise & Spence, 2012), and Conjoint Analysis (Ares & Deliza, 2010; Baptista et al., submitted; Gislason et al., 2020), as well as focus group research (Lunt, 1981; Rapaille, 2007; Stern, 1981) have all been used by those researchers wanting to establish the more abstract, symbolic/semiotic meanings that may be associated with specific abstract visual design features (typically when embedded in product packaging) (see Table 2 for a summary of techniques). We presumably also need to consider the benefits of the consumer neuroscience, or neuromarketing approaches to design (see also Huang et al., 2021). However, it should be noted that despite a longstanding interest in the consumer neuroscience of product packaging (see Weinstein, 1981, for early research), the body of research that has been published to date remains fairly limited (see Moya et al., 2020, for a review).

Having set the background for our consideration of the various meanings associated with abstract visual packaging design cues in the world of food and beverage packaging, we will now take a closer look at each of the main visual design features in turn, starting with perhaps the most frequently studied abstract visual design feature, namely colour.

### On the multiple meanings of packaging colour and other visual appearance cues

Perhaps the single most extensively studied visual design feature on product packaging is colour (Baptista et al., 2021; Crilly et al., 2004; Danger, 1968, 1987; Déribéré, 1978; Favre, 1968; Huang & Lu, 2013; Kovač et al., 2019; Labrecque & Milne, 2012, 2013; Labrecque et al., 2013; Merlo et al., 2018; Theben et al., 2020; Wheatley, 1973; see Spence & Velasco, 2018, for a review). Consider here only Coca-Cola’s dominant use of (and association with) the colour red (and rounded white text) which has been successfully linked to the brand and, by doing so, has seemingly managed to overcome any potential language/cultural barriers (Van Den Berg-Weitzel & Van Den Laar, 2001). The colour red and round typeface both also convey/prime notions of sweetness (Velasco et al., 2015b; Velasco et al., 2018a, b; Woods et al., 2016). However, in certain contexts red also acts as an indicator of temperature (i.e. warmth, think about the colour on taps; Ho et al., 2014, see Spence, 2020b, for a review) and can signal danger/prime avoidance motivation (Lunardo et al., 2021; cf. Labrecque & Milne, 2012), as well as attraction (Tham et al., 2020). In other words, a particular hue of product packaging may be associated with a range of attributes/qualities, and the extent to which any one of these different associations are primed may well depend on the context, or category, in which that colour is presented (Amsteus et al., 2015). Intriguingly, Coca-Cola’s main international competitor (Pepsi) rebranded some years ago, choosing the colour blue (Cooper, 1996), presumably to help distinguish itself within the cola beverage category (see also Baxter et al., 2018, on the importance of brand colour).

For further evidence of the learning of arbitrary associations between packaging colour scheme and flavour consider only the crisps/potato chips category, mentioned earlier (Piqueras-Fiszman et al., 2012). That said, there appears to be some degree of consistency with which different colours are used to signal different flavour variants. For instance, Velasco et al. (2015a) demonstrated that congruency (e.g. red/tomato), relative to incongruency (e.g. yellow/tomato), between the colours used in product packaging and flavour labels facilitated their participants’ visual search performance (as evidenced by reduced reaction times) for target crisp packets. Packaging colour is, then, sometimes used to signal variation within a category, whereas, at other times, it may be associated with a particular brand (and thus indirectly also with a category instead).

Occasionally, however, brands have deliberately chosen to contravene the colour code of the category. Take, for example, the use of blue packaging for cheese-and-onion flavour crisps, and green packaging for salt-and-vinegar, introduced by Walkers in the UK to try to secure exposure of customers to their new flavour variety (c. 1984; see Piqueras-Fiszman et al., 2012). This decision was apparently based on the notion that our shopping choices are, in large part, based on colour (see Spence & Velasco, 2018, for a review). Other crisp manufacturers in the UK had historically tagged salt-and-vinegar with blue. So, by packaging their new flavour variant (cheese and onion) in the well-establish blue of salt and vinegar, the idea was that consumers would shop by colour and hence be inadvertently exposed to a new flavour variant. Spence and Piqueras-Fiszman (2012) highlighted the example of a white wine that was called ‘Red’ and which had a bright red label, as an ultimately unsuccessful example of incongruency. Hence, sometimes abstract visual design features such as hue are chosen after considering both their ability to differentiate the product from others in the marketplace and the specific connotative meaning of the hue. The reader is referred to Labrecque and Milne (2013) for further discussion of colour norms and the benefits of colour differentiation in the marketplace (see Spence & Velasco, 2018; Vermeir & Roose, 2020, for reviews).

In addition to pink and red being associated with sweetness, Woods et al. (2016) demonstrated that white and blue were associated with saltiness, green and yellow with sourness, and black and green with bitterness. Consumers have also been shown to perceive a candy bar with a green label as being healthier than one with a red label, even when the caloric information on the labels happens to be identical (Schuldt, 2013). While the majority of the research that has been published to date has tended to focus on the colour of outer packaging, it is interesting to note that inner packaging colour has started to attract the attention of researchers, especially for those products such as individual yoghurt pots, where the consumer often consumes the product directly from the packaging (see van Esch et al., 2019; see also Krishna et al., 2017, on the importance of distinguishing between inner and outer packaging).

Taken together, the research that has been published to date highlights the multiple meanings that may be associated with a given colour in the context of food and beverage packaging. Given that packaging colour may be associated with one of a number of attributes including flavour (Piqueras-Fiszman et al., 2012), variant (Cutolo, 2021), brand (as in the case of signature colours; Baxter et al., 2018), but also more generally with other attributes such as healthfulness (Mai et al., 2016; Schuldt, 2013; Tijssen et al., 2017; see also Cavallo & Piqueras-Fiszman, 2017) and luxury/cheapness (see Velasco & Spence, 2019c; Wheatley, 1973; see also Hagtvedt, 2014; Huang & Lu, 2013; Spence & Velasco, 2019, for other examples), the relevant question becomes: Which of the many possible meanings dominates in the mind of the consumer in any given situation or context? It is worth noting that a problem with much of the laboratory/online research conducted to date is that the dimension of interest to researchers has often been presented to consumers in the response scale’s anchor labels. This is obviously unlike the conditions of everyday life, where the most salient dimension of meaning might well be determined by the aisle in a supermarket, or the category that the consumer is inspecting, or perhaps by the consumer’s current thoughts/objectives/goals (see Huang & Lu, 2015). Indeed, it is even possible that there may be a hierarchy of associations with some being dominant over others, again possibly depending on context.

Beyond hue, it is important to note how other visual appearance properties, such as lightness/saturation (Mai et al., 2016) and glossiness (De Kerpel et al., 2020; see Spence, 2021b, for a recent review) can also convey different messages/meanings when present on product packaging. For example, light and pale colours tend to be associated with healthfulness. That being said, as Mai et al. (2016) have noted, lightness may have different meanings for different people, and the association between lightness and perceived healthiness can be moderated by other factors including the goals of the consumer. Glossiness, on the other hand, tends to be associated with greasy and/or unhealthy foods by the majority of consumers (see Spence, 2021b, for a review).

It is at around this point that one might be tempted to ask, do visual design cues, such as colour, do anything more than merely set/prime a consumer’s expectations? And here, while online research that merely assesses expectations is just so much easier to conduct (e.g. Woods et al., 2015), nevertheless there are a few studies showing how changes to the visual appearance of the receptacle in which a product is packaged can significantly influence not just people’s expectations, but also their experience (cf. Carvalho & Spence, 2019). At the same time, however, it is important to note that the power of any visual cue, such as colour, as discussed in this section, to modulate taste is dependent not only on the strength or robustness of the association between the colour and the related taste, but also the degree of discrepancy between the consumer’s expectation and their actual experience (e.g. see Schifferstein, 2001; Spence & Piqueras-Fiszman, 2012, for reviews).

### Shape, packaging, and crossmodal correspondences

Given what we have seen so far, it should be clear that the shape of product packaging may convey (or prime) multiple distinct meanings to the consumer. Specific packaging shapes may be associated with quality, brand, gender, healthfulness, and strength (see Hine, 1995; Stern, 1981). And, just as for the case of colour, the various different theoretical accounts all have something to say regarding the meaning(s) of shape cues in product packaging (see Table 1). One recent area of interest amongst researchers has been on the crossmodal correspondences between shapes and taste/flavour (Velasco et al., 2016a, 2016b). The latest research has highlighted the fact that basic shape properties are associated with taste in a manner that can, at times, seem almost synaesthetic (Cytowic & Wood, 1982) though, importantly, is not (Deroy & Spence, 2013). Roundness, for example, tends to be associated with sweetness, whereas angularity tends to be associated with bitterness, sourness, and saltiness (Spence & Deroy, 2012, 2013). Sourness is also associated with asymmetrical, rather than with symmetrical, visual designs (see Salgado-Montejo et al., 2015a; Turoman et al., 2018). Given such findings, shape-taste correspondences can be incorporated into a range of design elements including everything from typeface (de Sousa et al., 2020; Mead et al., 2020; Velasco & Spence, 2019b; Wang et al., 2020) to lines and shapes on/of labels (Li et al., 2022; Matthews et al., 2019), transparent windows (Simmonds et al., 2019), and even the distinctive image moulds of specific packaging forms or silhouettes (Meyers, 1981; Overbeeke & Peters, 1991; Spence & Piqueras-Fiszman, 2012; Wang & Sun, 2006). While certain shapes are associated with specific flavours, atypical food packaging might attract attention and increase product salience (cf. van Ooijen et al., 2016). However, as the latter researchers point out, atypical packaging can also have a detrimental effect on the consumer’s product evaluation. Specifically, it can enhance the processing of product information which, in turn, decreases the persuasiveness of weak (i.e. unconvincing) messaging.

It is currently unclear what the basis of shape/taste associations might be (Dichter, 1971; Gal et al., 2007; Obrist et al., 2014; Spence & Deroy, 2012, 2013). According to one suggestion, it may simply be that pleasant shapes are linked with pleasant tastes (e.g. round with sweet) while potentially threatening stimuli (e.g. angular shapes and bitterness) may be grouped together. One can think of this as a kind of emotional mediation, or affective correspondence, account (Salgado-Montejo et al., 2015a). However, according to Obrist et al. (2014), roundness may be associated with sweetness because of the gradual change in taste sensation that is experienced with this kind of taste stimulus. Obrist et al. demonstrated that people typically experience sweetness as building slowly, having a rounded or smoothed peak, and then decaying slowly on the palate. By contrast, sour tastes are experienced as having a much sharper temporal onset and offset. That said, the fact that many crossmodal correspondences have been incorporated conventionally in product packaging for decades, means it is hard to discount the possibility that consumers have simply internalized (perhaps unconsciously) the regularities of the marketplace.

Cross-cultural research from Bremner et al. (2013) is of relevance here. These researchers investigated the Himba tribe in Namibia. These hunter-gatherers have no written language nor access to supermarkets. Intriguingly, this group does not show the same taste-shape correspondences that have been documented elsewhere. Specifically, they exhibited no association between angularity and carbonation in sparkling (vs. still) water (cf. Spence, 2019a). What is more, they associated milk chocolate (i.e. sweet) with angular shapes while matching dark chocolate (i.e. bitter) with round shapes—the opposite of what has been demonstrated repeatedly elsewhere. This suggests that the internalization of the visual communication conventions of the marketplace may well play an important role in explaining certain crossmodal correspondences relevant to product packaging (and/or product forms). Notice here how, should such idiosyncratic results be replicated, they would argue against Obrist et al.’s (2014) putative account of taste-shape correspondences. The various explanations (see Table 1) for the communicative function of shape cues should not, of course, be treated as mutually exclusive, and indeed several explanations have been shown to contribute to explaining a number of the crossmodal correspondences that have been documented in the literature to date (Spence, 2020a).

Shape may also be associated with health, strength, or possibly even with taste properties (Parise & Spence, 2012). There is also a literature on branded ‘image moulds’: That is, distinctive packaging forms or silhouettes (Arboleda & Arce-Lopera, 2015) that may become associated with a specific brand (e.g. consider only the contour of a Coca-Cola bottle; Prince, 1994) and/or with a specific class of product (Söderlund et al., 2017), as happened some years ago with the sloped-shouldered Wishbone salad dressing bottle (see Hine, 1995; Meyers, 1981). The suggestion is that the most successful packaging forms have become image moulds in lieu of the fact that the shape features (e.g. rounded or angular) are consistent with the key brand attributes (Anon., 1994; Gislason et al., 2020; Parise & Spence, 2012). On occasion, semantically meaningful shapes have been incorporated in packaging design (e.g. as in the successful case of the green tea sold in Japan in a green plastic bottle that itself resembles bamboo; see Visser, 2009, pp. 8–9).

Importantly, and just as was the case for colour (discussed earlier), given that packaging shapes are associated with a variety of different attributes, consumers may need to be primed to think about taste (gustation) before they discriminate between shapes as a function of taste. That is, consumer goals (or context) may be critical in terms of determining the communicative function of packaging shape. That said, and again, there may also be a hierarchy of values. Addressing these issues constitutes an important task for future applied packaging research. And, once again, future research might benefit from considering how Mukherjee et al.’s (2022) theory of semantic discriminability. In particular, it would be interesting to know more about the role of context, or comparison stimuli, in determining whether the concepts that are primed in the consumer’s mind by specific shape cues might not reflect inference rather than necessarily direction association.

### When orientation biases meaning

The orientation of abstract visual design features (such as shapes) on product packaging also matters when it comes to communicating with the consumer. For instance, people have been shown to respond very differently to triangles as a function of whether they happen to point upwards or downwards (Zhao et al., 2017, 2020). Triangles, or other angular shapes, that pointing downwards/towards the viewer can trigger a short-lasting neural fear response in the human amygdala (Larson et al., 2007; Watson et al., 2011). One explanation that has been put forward for this finding is that downward-pointing, relative to upward-pointing, triangles generate a change in visual processing that is driven by negative affective properties (Watson et al., 2011).

Meanwhile, lines ascending to the right have very different connotations than when the same line ascends to the left instead (see Spence et al., 2019, for a review). The former appear to be associated with positive dynamism, whereas the latter tend to have a much less positive connotation (see Velasco et al., 2015c). By way of example, Mead et al. (2020) reported that right-slanted fonts were effective in evoking thoughts of an advertising campaign that was moving forward (and thus that time was running out) which, in turn, influenced people’s purchasing intentions. Intriguingly, it has even been suggested that the response to oriented lines can appear almost innate (see Karim et al., 2016).

Notice here also how, depending on its orientation, the same curved line may look like a smile or a frown (Salgado-Montejo et al., 2015b). Even the direction in which individual faces are looking (i.e. to the left or right) has been shown to subtly prime different expectations/associations in the mind of the consumer (Park et al., 2021). Specifically, leftward-facing people are deemed to be ever-so-slightly more attractive which, in turn, has been shown to promote more positive attitudes towards products.

As another example, the customers in one intriguing study were invited to evaluate the ‘house blend’ of coffee (Van Rompay et al., 2019, based on work by Rorink, 2018). These authors established that horizontal vs. vertical stripes on a poster in a Dutch coffee shop influenced customers’ ratings of the coffee. Van Rompay et al. used the concept of ‘embodied cognition’ to help explain their findings. Specifically, their suggestion was that luxury and power are associated with ‘top-shelf’ and ‘looking down’ on others, respectively. These researchers reported that vertical, relative to horizontal, stripes positively influenced taste experience, quality perception, and purchase intention of coffee. The argument is that a vertically oriented advertising display may invoke perceptions of power (i.e. Machiels & Orth, 2017; Schubert, 2005; Sundar & Noseworthy, 2014; van Rompay et al., 2012). The suggestion was that this, in turn, caused the consumers to rate the coffee as having a more powerful/intense taste, relative to those in a horizontally oriented advertisement condition.

### Position implicitly conveys meaning

Researchers have explored other indicators of verticality, such as the positioning of elements on product packaging. For instance, Fenko et al. (2018) assessed the impact of incorporating an image of a lion (as a metaphor for strength) on a package of coffee beans. The lion could either appear at the top or bottom of the packaging. The lion’s location was shown to influence both multisensory flavour perception and purchase intentions. When the image was situated at the bottom of the packaging, the coffee was perceived to be stronger. Fenko and her colleagues argued that this is consistent with the theory of grounded cognition, whereby a ‘strong is heavy’ metaphor is activated, with heavy objects usually located on the ground. Similarly, Togawa et al. (2019) found that an image of a food item placed lower on the product packaging enhanced both people’s expectations and perceptions of the heaviness of the product’s flavour. Interestingly, the association between position and heaviness influenced consumers’ decisions regarding healthy eating, such that they consumed less of the ‘heavy’ food and tended to choose a healthier snack option instead.

In research exploring the association between healthiness and vertical position, Wang and Basso (2021) recently demonstrated that people associate healthy food (i.e. fruit salad) with high verticality, whereas unhealthy food (i.e. ice cream) was associated with low verticality instead. These researchers suggested that conceptual metaphor theory could be used to explain their findings in that health is commonly associated with ‘up’ (being upright; sayings such as ‘She is in peak physical condition’), while illness is associated with ‘down’ (being forced to lie down in bed; ‘She felt under the weather’ or being ‘down in the dumps’). Meanwhile, in an earlier study, Deng and Kahn (2009) reported that the consumer’s goals (e.g. to be healthy) influenced their preferences for the location of objects on product packaging. Specifically, those consumers with a health goal exhibited a weakened preference for packages where the image was situated at the bottom (i.e. heavy location). While the design features whose position has been varied were semantically meaningful stimuli in the above-mentioned cases, it might be expected that similar associations would be documented were it to be the position of an abstract visual design element that was varying instead.

Elsewhere, Simmonds et al. (2018b) demonstrated that the left/right position of transparent windows embedded in product packaging significantly influenced ratings of a range of product qualities (e.g. overall liking, quality, willingness to purchase) for fake brands of lemon mousse, cereal, and chocolate. Finally here, mention should be made of Salgado-Montejo et al. (2015b) study highlighting how the position of a concave/convex line on the front of product packaging (top, middle, or bottom) biased the likelihood with which that design feature was interpreted as a smile. Specifically, the line was more likely to be interpreted as a smile when it appeared at the bottom, rather than the top, of product packaging, thus suggesting a degree of anthropomorphism. Note here that anthropomorphism in product/packaging design tends to increase consumer preference (Batra et al., 2016). Similar benefits have now been noted across a wide range of product categories (e.g. Rapaille, 2007; Wang & Basso, 2019).

### Interim summary

An emerging body of scientific research has started to document the various meanings that are associated (by consumers) with specific visual cues/design features in product packaging in the food and beverage category. Colours (and saturation, lightness, and finish/glossiness) on product packaging have all been associated with various taste/flavour properties, product quality, and the healthiness of the product contained within the packaging. Stripes, be they vertical or horizontal, represent an interesting class of design feature in not having a clear connotative meaning (Albertazzi et al., 2021; Walker & Walker, 2012) established in the literature to date. In contrast to other design features mentioned so far, stripes represent an abstract visual design feature that has (to date at least) seemingly received little research attention from those interested in product packaging (see, for example, the absence of coverage in Velasco & Spence, 2019a), despite various companies choosing to introduce stripes in their product packaging.

## Combining abstract visual design features

Having reviewed the evidence concerning the meaning of individual abstract visual design cues, such as colour, shape, and orientation in product packaging, it seems worthwhile turning to the question of how various combinations of abstract visual design cues may be interpreted by the consumer. This can either be combinations of colours, as in colour pairs or triplets, or combinations of different visual features, such as the combination of colour and shape. However, given the combinatorial explosion that one is soon faced with when combining different visual design features, our focus in this section will be on the associations/meaning that may be associated with, or primed by stripes, given their neglect in the literature on crossmodal correspondences to date, together with their frequent appearance in nature and product packaging.

The use of stripes introduces combinations of visual features such as colour pairs which, in turn, might be expected to generate interesting effects such as colour contrast. The meaning of colour pairs has been well-studied but depends, to a certain degree, on the specific relation between the component parts. For example, side-by-side vs. foreground/background arrangements will need to be considered by package designers, and even which element is in the fore-/back-ground (Woods & Spence, 2016; Woods et al., 2016; cf. Deng et al., 2010; Schifferstein & Howell, 2015). Pink on a white background, for example, is more strongly associated with sweetness than (a) when either colour is presented in isolation, (b) when white is presented against a pink background, or (c) when these colours are presented side-by-side instead.

There is a longstanding, separate literature on colour-shape correspondences (e.g. Dreklser & Spence, 2019). The research has demonstrated that combinations of colour and form sometimes take on specific symbolic (i.e. the image/association that comes to mind with respect to a product; Kujala & Nurkka, 2012) and/or affective (i.e. the emotion elicited by a stimulus) meaning (Ares & Deliza, 2010; Kaeppler, 2018; Oyama, 2003; Spence, 2021c). One might question whether cues are combined based on similar connotative meanings, as assessed by approaches such as the semantic differential technique (Osgood et al., 1957; Snider & Osgood, 1969; cf. Henson et al., 2006; Kawachi et al., 2011; Morich, 1981; Oyama et al., 1998; Schaefer & Rotte, 2010; Suzuki et al., 2005). Consider, for example, how red and highly angular shapes often co-occur (e.g. on the front of beer cans; Spence, 2012). This constellation of abstract visual design features may go particularly well together because, when presented individually, both stimuli are associated with activity and dominance (rather than with passivity and submissiveness) according to semantic differential analysis (Adams & Osgood, 1973).

Ensuring the congruency^6^ of different visual design elements has been suggested to be an important part of successful design (Fürst et al., 2021; Heatherly et al., 2019; Matthews et al., 2019; Salgado-Montejo et al., 2014), processing fluency,^7^ and effective visual search (Velasco et al., 2015a). From a marketing perspective, the wrong combination of design features can exert a drastic negative impact on brand perception and, importantly, sales. Tom et al. (1987) provide an example where a Swiss coffee brand redesigned their packaging. Although the new packaging won awards for design, sales plummeted. The problem appeared to be that diagonal stripes of mauve were simply not deemed congruent with the conventions of the category (i.e. coffee packaging) by the consumer. Favre and November (1979) provided several other historic examples of unsuccessful packaging colour rebrands. Hence, having established the connotative meaning of specific visual design features as a function of their position/orientation, manufacturers have a choice to either follow the conventions of the category or go for something different. However, only some brands seem able to carry-off incongruent signalling in the marketplace (cf. Sundar & Noseworthy, 2016), especially given the disruption to processing fluency that such incongruency is likely to elicit (Lunardo & Livat, 2016; cf. Herrmann et al., 2013; Labroo et al., 2008). Wheatley (1973) gives the example of the hugely successful Alpen muesli that came out with matte black packaging for their muesli in the 1970s in a mostly white and sunny yellow coloured product category (i.e. breakfast cereal). More recently, several fabric conditioner brands have similarly attempted to disrupt the colour conventions of the laundry category by again coming out with black packaging in a mostly white and blue packaging colour category. It is interesting to consider here how the desire to stand out on the shelf, and so capture the customer’s visual attention more effectively (see Reutskaja et al., 2011; Spence & Piqueras-Fiszman, 2012), often leads to the colour (and other visual design) conventions of the category being overturned. This strategy has been used very effectively in recent years in the drinks category, by those such as Gatorade, and more recently, Innocent (the latter with their Bolt from the Blue product launch; see Spence, 2021d).

Certain combinations of shapes and colours may take on symbolic or semantic associations. Think, for example, of how a red circle or plus sign on a field of white may prime notions of the Japanese flag and the Red Cross, respectively (Chen et al., 2021). One might also consider the semantic meaning of the iconic Lucky Strike cigarette packaging showing a red circle against a white background (designed by Raymond Loewy). Furthermore, people typically associate yellow with a crescent shape, presumably because they are reminded of the moon (Dreksler & Spence, 2019; Woods et al., 2013). It is worth noting that combinations of colours in stripes can be associated with a particular (semantic) meaning which is, at times, dependent on the orientation of the stripes (see below).

### Recent commercial examples: on the use and orientation of coloured stripes on product packaging

Given Kentucky Fried Chicken’s (KFC’s) recent decision to update the design of their food product packaging (Anon., 2021) and stores (Valinsky, 2020) to emphasize their signature vertical red-and-white stripes, we have chosen to use them as a recent commercial example regarding the use of stripes in product branding and how these design elements contribute to perception. Similarly, Devondale—a company offering a range of dairy products—updated their packaging back in 2012 such that it included horizontal light blue-and-white stripes (Hicks, 2012). The branding on this iconic Australian range of dairy products is reminiscent of the famous ‘Cornishware’ style of English kitchen pottery. Of relevance, given Devondale’s use of blue-and-white stripes, and the fact that the company has ties to dairy farming, this design may also be intended to evoke thoughts of farms, cottages, and cows (see also Rodionova, 2016, for supermarkets attempting to create associations by using fake farm names).

Note here how the semantic/affective associations with horizontal light blue-and-white stripes cannot simply be predicted based on the consumer’s response to the individual abstract visual design cues (Spence et al., 2015a, b). One might consider whether the blue caps on traditional milk bottles could also provide a basis for the use of this combination of colours (i.e. blue cap plus white milk). Abstract patches of blue and white, when presented together, are associated with a salty taste (Woods & Spence, 2016; Woods et al., 2016). It is, though, worth noting that the participants in Woods and colleagues’ online research were primed to think in terms of the associations between colour pairs and basic tastes, given that they were forced to choose between the basic tastes when responding (cf. Mukherjee et al., 2022). Thus, even though the combination of blue and white may be more strongly associated with salty than with other tastes, that does not preclude the possibility that the consumer might be primed to think of milk/dairy more than they are to think of salt on seeing this combination of colours.

Similarly, a particular shade of purple, red, or turquoise might well be expected to prime associations with the branded colours of Cadbury’s chocolate, Coca-Cola, and Tiffany jewellery, respectively, more than with specific taste qualities (see Baxter et al., 2018). It would be helpful if future research, in which the associations primed in consumers by viewing specific combinations of colours, were not constrained by a forced-choice design (e.g. as in the open responding required in the Word Association task, for example; Piqueras-Fiszman et al., 2013). At the same time, given the multitude of responses that might ensue, it would perhaps help to give the consumer a particular context (e.g. ‘What comes to mind if you saw this particular combination of colours in the refrigerated section of a supermarket?’). A third example of the use of coloured stripes in product packing relates to the LGBTQI + movement and the incorporation of rainbow stripes into product design (e.g. Ralph Lauren t-shirts, ADIDAS shoes; see Yates, 2021, for a number of other examples). By way of example, the incorporation of the LGBTQI + flag, which combines five colours, into product design may have implications for the connotative meaning and brand perception beyond the associations primed by colours.

In relation to KFC and Devondale, products in warm-coloured packaging (e.g. red) are deemed to be less healthy than are those presented in packages using cooler colours (e.g. blue; see Singh, 2006; Van Rompay et al., 2016). Woods et al. (2016) demonstrated that colour pairs communicate basic tastes and found that, for example, the pairing of white and red was associated with saltiness. This is interesting considering KFC’s recent rebrand where the red-and-white stripes were made more vibrant. Woods et al. also reported that the combination of white and blue better portrayed saltiness than when using either colour alone; previous research had shown that each colour was associated with saltiness (Favre & November, 1979; Spence et al., 2015b; cf. Velasco et al., 2016a, 2016b). It might seem odd then that Devondale should choose to use this pairing on dairy products as, although butter is often salted (but can also be unsalted), a company might want to avoid generating an expectation of salty milk. Perhaps the hope is to generate mental imagery associated with Cornishware in those who happen to be familiar with this famous traditional style of pottery from the UK, and that this will override the blue-white/saltiness correspondence (at least in those who are familiar with Cornishware).

A separate literature has explored the perceptual differences generated by lines as a function of whether they are shown horizontally vs. vertically (Avery & Day, 1969). People tend to perceive horizontal lines as being shorter than lines of equivalent length presented vertically. Such visual illusions have implications for the form (or orientation) of product packaging in that consumers perceive short, wide packages to hold less volume than tall, slender packages (see Chen & Shi, 2017; see also Raghubir & Greenleaf, 2006); Raghubir & Krishna, 1999; cf. Cheskin, 1951, pp. 193–194). Think, here, only of Piaget’s conservation task (Piaget, 1952). To add another layer of complexity to this issue, the ‘Helmholtz Square’ illusion shows that a square comprised of vertical stripes appears to be shorter and wider than an identical square comprising horizontal stripes (Coren & Girgus, 1978; Seriously Science, 2014; Thompson & Mikellidou, 2009). In all the above cases, while the shape itself does not change, simply altering the orientation leads to a predictable change in visual perception. Interestingly, this may be of benefit to Devondale in their marketing of butter which is presented in rectangular containers. Although speculative, the Devondale container with its horizontal stripes might make the container look taller, thus creating an illusion such that people unconsciously believe they are getting more for their money.

Thus, the orientation of visual design elements on product packaging, and even the position of packaging on shelving (see Sunaga et al., 2016, on the lightness-elevation correspondence that can be used to guide shelf positioning), influences perception by priming those attributes that happen to be linked to specific visual design features. Hence, KFC which achieved success via the introduction of vertical red-and-white stripes (see Anon., 2021), and Devondale who use horizontal blue-and-white stripes on their brand packaging, may succeed independently of one another due to the influence of several, independent factors helping to determine/constitute the meaning of coloured stripes.

#### Assessing the effectiveness of stripes in product packaging

Evolutionary theory provides a possible, if highly speculative, explanatory framework for the success/appeal of stripes in the marketing of products in the food and beverage category (see above for the evolutionary account of several other visual design features). Although it is beyond the scope of this narrative review to comprehensively list all of the hypotheses relating to communicating signals, we outline a few particularly relevant ones below. The first thing to point out is that repetitious patterns (e.g. stripes) are common in nature—think of the zebra, zebra fish, or tiger snake, as examples (Coborn, 1991; Lieske & Myers, 1994). Repetitive patterns may have evolved in nature to stimulate ‘the receiver regardless of the position of the signal’ (Kenward et al., 2004, p. 412) on the retina. At the same time, however, the incorporation of stripes may serve somewhat different functions in different species. The zebra’s distinctive stripes, for example, help to deter flies from biting them (see How et al., 2020), while tigers might have stripes to hide/for camouflage, and bees perhaps to warn off other creatures (though see Stelzer et al., 2010, for evidence questioning the latter suggestion). In other words, the effect of stripes might rely on the qualities of the stripes, the combination of colours used, and the ecological niche inhabited by the animal.

In much the same way, it has been suggested that the presence of stripes might be used for camouflage or capture attention, colour is important both as an aid to foraging (Foroni et al., 2016)—though researchers argue about whether it developed to facilitate frugivory or folivory (e.g. Sumner & Mollon, 2003)—as well as a potential signal for mating/conspecific communication (e.g. Changizi et al., 2006; see also Humphrey, 1976, on the complex evolutionary meanings associated with colour). Hence, evolutionary accounts are currently both limited in the range of visual design cues that they can potentially provide an explanation for, and are often open to other interpretations (both evolutionary and otherwise), meaning that they are of only limited explanatory validity in decoding the visual aspects of packaging design.

Repetitive patterns such as stripes may be used in marketing because images created on the retina can vary in orientation as well as in position. Think, for example, of a shopper in a supermarket moving past a product from right-to-left, and then from left-to-right. This creates image sequences that are mirror reflections of each other. Stripes will be invariant when reflected (i.e. symmetrical), so the use of stripes may contribute to enhanced processing fluency (cf. Bigoin-Gagnan & Lacoste-Badie, 2018). Manufacturers of sour products may want to avoid the use of stripes though, given that sourness tends to be associated with visual designs that are asymmetrical (Salgado-Montejo et al., 2015a; Turoman et al., 2018). Remember here how a lack of congruency between visual design elements and expected taste attributes can negatively impact product attitudes (see Ares & Deliza, 2010). At the same time, however, it is worth noting that symmetry is processed fluently, and hence tends to be preferred visually (Pecchinenda et al., 2014).

One of the problems with products on supermarket shelves is the need to stand out when parts of the packaging may be obscured. Importantly, repetitive patterns such as stripes may be useful because they look similar even when parts of the product (or animal in nature) are obscured and, as such, will still be recognizable to an onlooker (Kenward et al., 2004). An important physiological process explaining the usefulness of stripes might be lateral inhibition. Lateral inhibition is defined as ‘the capacity of excited neurons to reduce the activity of their neighbours’ (Cohen, 2011, p. 1437). Lateral inhibition helps to enhance edges, and ‘makes it easier to distinguish objects from backgrounds under varying light conditions’ (Kenward et al., 2004, p. 415). As such, stripes have a greater apparent maximum intensity than do solid blocks of colour, and thus they tend to ‘pop out’. As brands have very limited time to attract the attention of potential buyers (Reutskaja et al., 2011; Sugermeyer, 2021), stripes might work well amongst the myriad products and advertising clutter on shelves. At a psychological level, one might also choose to invoke Gestalt theory to help describe the factors affecting the grouping of elements, such as lines, in product packaging (Ellis, 1938; Wagemans, 2015). Grouping principles such as grouping-by-similarity, grouping-by-proximity, and good continuation may sometimes also help to predict/explain why visual design features, such as stripes, are grouped in certain ways.

Finally, it is worth noting that stripes may also have other semantic associations, that have been built up through experience, and which may help to explain their meaning to consumers (i.e. independent of any specific evolutionary account). Consumers might, for instance, be primed by the sight of black and white stripes to think of prison uniforms, or perhaps a fashion icon such as Coco Chanel, or a sports team (e.g. Juventus). There are, in other words, likely always going to be a range of explanations behind the ‘meaning(s)’, or associations, that happen to be primed by any given visual design feature. It is important to note that the various explanations should not be treated as mutually exclusive, and indeed several explanations have recently been shown to contribute to explaining many of the crossmodal correspondences that have been documented in the literature (Spence, 2020a).

### Interim summary

The research reported in this section highlight how the meaning attributed by consumers to the combination of different abstract visual cues, such as colour pairs or colour and shape, typically cannot simply be predicted simply on the basis of the consumers’ response to the individual visual cues when assessed in isolation. Sometimes, for example, specific combinations of visual design cues may deliver a configuration that takes on a meaning of its own, as with the thick horizontal blue and white stripes that may be associated with Cornishware pottery, while the individual colours are likely to be associated with a salty taste (see Spence et al., 2015a, b). By contrast, the red and white vertical stripes of KFC packaging might be expected to cue saltiness and power, possibly enhancing the taste of the product (cf. Fenko et al., 2016). One of the important areas for future research on the visual design of product packaging is therefore to understand more about the meaning to consumers of various combinations of abstract visual design cues (e.g. such as the combination involved in coloured stripes).

## Conclusions and future directions

The majority of the research on the visual design of product packaging has addressed individual visual design features. However, while this is undoubtedly a fruitful first step, it is crucial to note that any realistic example of food or beverage packaging will inevitably incorporate several visual design elements (see Favre & November, 1979; Hine, 1995; Visser, 2009). Hence, the question immediately becomes one of whether it is possible to predict the consumer’s response to the combination based simply on how they respond to individual abstract visual cues, such as colour or shape/form (Labrecque et al., 2013). The limited evidence that has been published to date certainly suggests that while abstract visual design elements that are congruent in terms of their connotative meaning, and/or that are linked by their crossmodal correspondence, are sometimes combined, there are other situations in which a specific configuration of visual design cues takes on a semantic meaning that goes beyond the meaning of the individual cues (Dreksler & Spence, 2019; Matthews et al., 2019; Spence, 2020a; Zhao et al., 2020; and see Velasco et al., 2014a, for a review). It should, of course, further be remembered that visual design cues are but one element of multisensory packaging design.

Furthermore, although several studies have shown that individual visual design features (e.g. colours) have similar meanings across cultures (Adams & Osgood, 1973; Wheatley, 1973), some of the meanings (or codes) of packaging would appear to be market specific (Velasco et al., 2014b). This is obviously an important area for future research as far as international brands are concerned. However, returning to a point we made a moment ago, there is currently very limited evidence assessing whether combinations of features influence consumers from different cultures in similar ways (see Van Doorn et al., 2017, for one example relating to the influence of the height and width of coffee cups). One intriguing recent approach to establishing the affective or connotative colour associations has been based on machine learning (e.g. Jahanian et al., 2017; Jonauskaite et al., 2019b; see also Schloss et al., 2019).

At times, of course, design features are incorporated to make products stand out, and thus facilitate visual search for product packaging (e.g. Jansson, Marlow, & Bristow, 2004; Shen et al., 2015; Velasco et al., 2015a; Zhao et al., 2017). Importantly, this can help to facilitate information processing (van Ooijen et al., 2016) but, depending on how design features are integrated, also has the potential to negatively impact product evaluations (Spence & Piqueras-Fiszman, 2012; Sundar & Noseworthy, 2016). There is also a growing awareness that certain visual designs that have been shown to work well in the setting of physical bricks and mortar store may need to be modified/simplified to maximize their appeal for the online shopping setting (Reinoso-Carvalho et al., 2021).

As our understanding of the meaning, or connotation, of visual design elements of product packaging in the food and beverage category continues to grow, based on the theory of crossmodal correspondences, there is an opportunity to predictively develop packaging that has been optimized to combine visual features such as colour, shape, orientation, and position in order to convey the appropriate meaning (Jacquot et al., 2016; Velasco et al., 2014a; see Spence, 2020a, for a review) and/or capture the consumer’s attention. On occasion, visual design elements may be combined in an attempt to capture the consumer’s attention (see Piqueras-Fiszman et al., 2012) but, given the likely loss of processing fluency (Labroo et al., 2008; cf. Dohle & Siegrist, 2014), this technique should be used cautiously. Of course, any change in product/packaging design may lead to success simply because it is novel and/or captures the shopper’s visual attention (as in the case of the Bolt from the Blue from Innocent Drinks; Spence, 2021d). However, altering iconic visual designs can all-too-easily lead to a backlash from consumers that can adversely affect sales. PepsiCo discovered this some years ago when they changed their iconic ‘straw in a juicy orange’ design on their Tropicana packaging (Airey, 2010; Marion, 2015; see also Favre & November, 1979).^8^

As highlighted by this review, the scientific approach to visual design of food and beverage product packaging is rapidly contributing knowledge in this field, and helping product designers/marketers to significantly increase sales (Sugermeyer, 2021). The emerging understanding of the connotative meaning/crossmodal correspondences that are associated with specific abstract visual design cues, such as colour, shape, orientation, and position means that it is increasingly possible to predictively prime certain attributes. At the same time, however, most product packaging incorporates a variety of design elements, and their meaning, in combination, is not always easy to predict from elements studied in isolation. There is, therefore, a danger of combinatorial explosion should one try to map out the meaning of a wide array of combinations of design features. At the same time, as should have become apparent from the above discussion, researchers and practitioners still remain a long way from developing a commonly agreed account of visual design. Perhaps, though, this should not come as any surprise, given the variety of signs and contexts evoked by the visual design of food and beverage, or for that matter, any other category, of packaging.

## Data Availability

Not applicable.

## Footnotes

1 Synaesthesia refers to the phenomenon whereby stimulating one sense (or sensory dimension) leads to automatic, involuntary, and idiosyncratic perceptual experiences in a second sense, or sensory dimension (cf. Oyama et al., 1998).

2 Note that these terms are used scientifically, as opposed to colloquially, usage. As such taste is used to refer to one of the gustatorily determined basic tastes (e.g. sweet, sour, bitter, salty, sour, and umami), whereas flavour refers to the combined experience of taste and smell as in the experience of citrus, fruity, floral, or herbal notes (see Spence et al., 2015a, b).

3 Abstract visual design features/properties in packaging design include any simple feature (such as a colour, shape, and visual texture) that does not have an obvious semantic meaning. Note that while signature hues associated with brands might well be said to represent a simple design feature that has become imbued with semantic meaning (i.e. whatever the consumer associates with the brand, e.g. Baxter et al., 2018), such individual hues, along with other specific colours, will be treated as abstract visual design features here.

4 According to Lafontaine et al. (2020, p. 244): ‘Associative learning is defined as learning about the relationship between two separate stimuli, where the stimuli might range from concrete objects and events to abstract concepts, such as time, location, context, or categories’.

5 Here, already, one might start to wonder whether inferred colour-concept relations (see Tham et al., 2020) are as effective in terms of consumer perception/behaviour as the inferred mappings that are presumably often picked-up by the research (see Spence & Levitan, 2022).

6 While congruency is a challenging notion to define, it is generally taken to refer to combinations of features or attributes that are perceived as ‘going well together’, possibly because the elements commonly co-occur, or because they share perceptual affinity/similarity (Amsellem & Ohla, 2016; though see Schifferstein & Verlegh, 1996).

7 The widely used notion of ‘processing fluency’ refers to the ease with which a given stimulus configuration can be processed. Processing fluency tends to be higher for those stimuli that are familiar, easy to process, and where the component stimuli are congruent. Processing fluency is positively valenced (Lunardo & Livat, 2016; cf. Labroo et al., 2008).

8 One might consider this slightly ironic given that Tropicana source their oranges from Florida where, traditionally, most oranges were the green-skinned variety (see Hisano, 2019).
